# Student’s Guide to the Diseases of the Eye

**Published:** 1884-10

**Authors:** 


Students' Guide to Diseases of the Eye. *By* Edward Nettleship, F. R. C. S. *Ophthalmic Surgeon to St. Thomas' Hospital, and to The Hospital for Sick Children, Great Ormond Street. Second American from the Second Revised and Enlarged English Edition. With a Chapter on Examination for Color Perception, by* William Thomson, M. D. *Professor of Ophthalmology in the Jefferson Medical College. Cloth, pp. 416. Philadelphia:* Henry C. Lea's Son & Co.

This is a carefully prepared little volume, likely to meet the demand of American students, who are so overburdened with lectures that there remains but little time for a thorough study of the so-called special branches. In the division of subjects, the author has deviated from the plan mapped out in the majority of text-books. The first chapter is a concise and comprehensive digest of "the means of diagnosis." The mind of the student is thus at the very beginning imbued with the importance of close and systematic observations.

The method advocated for the detection of faulty color perception is quite simple, and possesses some advantages over older ones. The one referred to has been adopted by the Pennsylvania Railroad for the examination of its employés. The various colors are numbered, thus permitting an expert at a distance to decide upon the results elicited and tabulated by a non-professional. The test skins have received odd numbers, the confusion colors even numbers. The value of the book is furthermore enhanced by a tabulation of the most important data relating to the connection between ophthalmic complaints and diseases of other organs of the body. The appendix, containing the principal formulæ employed by oculists, with brief references to the physiological action of mydriatis and myotics, is quite an important addition. B. B.

